# Supplementary material for: Limited dishevelled/Axin oligomerization determines efficiency of Wnt/β-catenin signal transduction
Source: eLife. 2020 Apr 16;9:e55015. doi: 10.7554/eLife.55015 (PMC7200158; doi:10.7554/eLife.55015)
Supplement: Supplementary file 1. — Nucleotide sequences for constructs discussed in this paper. There are two classes of plasmids: those used to make recombinant protein in bacteria in the pCDF MBP-TEV or pGEX-TEV vector backbone, and those used to express full-length Dvl proteins in cells for signaling assays, which are in the pCS2+ vector backbone under the control of the SP6 promoter. The full sequence of each vector backbone is provided, including appropriate sequencing primers for each vector backbone. For analogous constructs between mammalian and bacterial expression, please refer to the bacterial expression constructs for the exact nucleotide sequence. Yellow highlights indicate either the mutated residue or extra sequences inserted inside the native open reading frame. [file elife-55015-supp1.docx]

**Plasmid sequences**

Nucleotide sequences for constructs discussed in this paper. There are two classes of plasmids: those used to make recombinant protein in bacteria in the pCDF MBP-TEV or pGEX-TEV vector backbone, and those used to express full-length Dvl proteins in cells for signaling assays, which are in the pCS2+ vector backbone under the control of the SP6 promoter. The full sequence of each vector backbone is provided, including appropriate sequencing primers for each vector backbone. For analogous constructs between mammalian and bacterial expression, please refer to the bacterial expression constructs for the exact nucleotide sequence. Yellow highlights indicate either the mutated residue or extra sequences inserted inside the native open reading frame.

| *Construct ID* | | *Description* | *Vector backbone* | *ORF Sequence and/or associated PCR primer oligonucleotides* |  |
| --- | --- | --- | --- | --- | --- |
| pCDFDuet His6-MBP-TEV  Sequence with Mal-E (forward), T7-Term (reverse), or DuetDOWN1 (reverse)  GGGGAATTGTGAGCGGATAACAATTCCCCTGTAGAAATAATTTTGTTTAACTTTAATAAGGAGATATACCATGGGCAGCAGCCATCACCATCATCACCACGGTTCTTCTATGAAAATCGAAGAAGGTAAACTGGTAATCTGGATTAACGGCGATAAAGGCTATAACGGTCTCGCTGAAGTCGGTAAGAAATTCGAGAAAGATACCGGAATTAAAGTCACCGTTGAGCATCCGGATAAACTGGAAGAGAAATTCCCACAGGTTGCGGCAACTGGCGATGGCCCTGACATTATCTTCTGGGCACACGACCGCTTTGGTGGCTACGCTCAATCTGGCCTGTTGGCTGAAATCACCCCGGACAAAGCGTTCCAGGACAAGCTGTATCCGTTTACCTGGGATGCCGTACGTTACAACGGCAAGCTGATTGCTTACCCGATCGCTGTTGAAGCGTTATCGCTGATTTATAACAAAGATCTGCTGCCGAACCCGCCAAAAACCTGGGAAGAGATCCCGGCGCTGGATAAAGAACTGAAAGCGAAAGGTAAGAGCGCGCTGATGTTCAACCTGCAAGAACCGTACTTCACCTGGCCGCTGATTGCTGCTGACGGGGGTTATGCGTTCAAGTATGAAAACGGCAAGTACGACATTAAAGACGTGGGCGTGGATAACGCTGGCGCGAAAGCGGGTCTGACCTTCCTGGTTGACCTGATTAAAAACAAACACATGAATGCAGACACCGATTACTCCATCGCAGAAGCTGCCTTTAATAAAGGCGAAACAGCGATGACCATCAACGGCCCGTGGGCATGGTCCAACATCGACACCAGCAAAGTGAATTATGGTGTAACGGTACTGCCGACCTTCAAGGGTCAACCATCCAAACCGTTCGTTGGCGTGCTGAGCGCAGGTATTAACGCCGCCAGTCCGAACAAAGAGCTGGCAAAAGAGTTCCTCGAAAACTATCTGCTGACTGATGAAGGTCTGGAAGCGGTTAATAAAGACAAACCGCTGGGTGCCGTAGCGCTGAAGTCTTACGAGGAAGAGTTGGCGAAAGATCCACGTATTGCCGCCACTATGGAAAACGCCCAGAAAGGTGAAATCATGCCGAACATCCCGCAGATGTCCGCTTTCTGGTATGCCGTGCGTACTGCGGTGATCAACGCCGCCAGCGGTCGTCAGACTGTCGATGAAGCCCTGAAAGACGCGCAGACTAATTCGAGCTCGAACAACAACAACAATAACAATAACAACAACCTCGGGCCGGGTGCGGAAAACCTGTACTTTCAGGGATCCGTCGACAAGCTTGCGGCCGCATAATGCTTAAGTCGAACAGAAAGTAATCGTATTGTACACGGCCGCATAATCGAAATTAATACGACTCACTATAGGGGAATTGTGAGCGGATAACAATTCCCCATCTTAGTATATTAGTTAAGTATAAGAAGGAGATATACATATGGCAGATCTCAATTGGATATCGGCCGGCCACGCGATCGCTGACGTCGGTACCCTCGAGTCTGGTAAAGAAACCGCTGCTGCGAAATTTGAACGCCAGCACATGGACTCGTCTACTAGCGCAGCTTAATTAACCTAGGCTGCTGCCACCGCTGAGCAATAACTAGCATAACCCCTTGGGGCCTCTAAACGGGTCTTGAGGGGTTTTTTGCTGAAACCTCAGGCATTTGAGAAGCACACGGTCACACTGCTTCCGGTAGTCAATAAACCGGTAAACCAGCAATAGACATAAGCGGCTATTTAACGACCCTGCCCTGAACCGACGACCGGGTCATCGTGGCCGGATCTTGCGGCCCCTCGGCTTGAACGAATTGTTAGACATTATTTGCCGACTACCTTGGTGATCTCGCCTTTCACGTAGTGGACAAATTCTTCCAACTGATCTGCGCGCGAGGCCAAGCGATCTTCTTCTTGTCCAAGATAAGCCTGTCTAGCTTCAAGTATGACGGGCTGATACTGGGCCGGCAGGCGCTCCATTGCCCAGTCGGCAGCGACATCCTTCGGCGCGATTTTGCCGGTTACTGCGCTGTACCAAATGCGGGACAACGTAAGCACTACATTTCGCTCATCGCCAGCCCAGTCGGGCGGCGAGTTCCATAGCGTTAAGGTTTCATTTAGCGCCTCAAATAGATCCTGTTCAGGAACCGGATCAAAGAGTTCCTCCGCCGCTGGACCTACCAAGGCAACGCTATGTTCTCTTGCTTTTGTCAGCAAGATAGCCAGATCAATGTCGATCGTGGCTGGCTCGAAGATACCTGCAAGAATGTCATTGCGCTGCCATTCTCCAAATTGCAGTTCGCGCTTAGCTGGATAACGCCACGGAATGATGTCGTCGTGCACAACAATGGTGACTTCTACAGCGCGGAGAATCTCGCTCTCTCCAGGGGAAGCCGAAGTTTCCAAAAGGTCGTTGATCAAAGCTCGCCGCGTTGTTTCATCAAGCCTTACGGTCACCGTAACCAGCAAATCAATATCACTGTGTGGCTTCAGGCCGCCATCCACTGCGGAGCCGTACAAATGTACGGCCAGCAACGTCGGTTCGAGATGGCGCTCGATGACGCCAACTACCTCTGATAGTTGAGTCGATACTTCGGCGATCACCGCTTCCCTCATACTCTTCCTTTTTCAATATTATTGAAGCATTTATCAGGGTTATTGTCTCATGAGCGGATACATATTTGAATGTATTTAGAAAAATAAACAAATAGCTAGCTCACTCGGTCGCTACGCTCCGGGCGTGAGACTGCGGCGGGCGCTGCGGACACATACAAAGTTACCCACAGATTCCGTGGATAAGCAGGGGACTAACATGTGAGGCAAAACAGCAGGGCCGCGCCGGTGGCGTTTTTCCATAGGCTCCGCCCTCCTGCCAGAGTTCACATAAACAGACGCTTTTCCGGTGCATCTGTGGGAGCCGTGAGGCTCAACCATGAATCTGACAGTACGGGCGAAACCCGACAGGACTTAAAGATCCCCACCGTTTCCGGCGGGTCGCTCCCTCTTGCGCTCTCCTGTTCCGACCCTGCCGTTTACCGGATACCTGTTCCGCCTTTCTCCCTTACGGGAAGTGTGGCGCTTTCTCATAGCTCACACACTGGTATCTCGGCTCGGTGTAGGTCGTTCGCTCCAAGCTGGGCTGTAAGCAAGAACTCCCCGTTCAGCCCGACTGCTGCGCCTTATCCGGTAACTGTTCACTTGAGTCCAACCCGGAAAAGCACGGTAAAACGCCACTGGCAGCAGCCATTGGTAACTGGGAGTTCGCAGAGGATTTGTTTAGCTAAACACGCGGTTGCTCTTGAAGTGTGCGCCAAAGTCCGGCTACACTGGAAGGACAGATTTGGTTGCTGTGCTCTGCGAAAGCCAGTTACCACGGTTAAGCAGTTCCCCAACTGACTTAACCTTCGATCAAACCACCTCCCCAGGTGGTTTTTTCGTTTACAGGGCAAAAGATTACGCGCAGAAAAAAAGGATCTCAAGAAGATCCTTTGATCTTTTCTACTGAACCGCTCTAGATTTCAGTGCAATTTATCTCTTCAAATGTAGCACCTGAAGTCAGCCCCATACGATATAAGTTGTAATTCTCATGTTAGTCATGCCCCGCGCCCACCGGAAGGAGCTGACTGGGTTGAAGGCTCTCAAGGGCATCGGTCGAGATCCCGGTGCCTAATGAGTGAGCTAACTTACATTAATTGCGTTGCGCTCACTGCCCGCTTTCCAGTCGGGAAACCTGTCGTGCCAGCTGCATTAATGAATCGGCCAACGCGCGGGGAGAGGCGGTTTGCGTATTGGGCGCCAGGGTGGTTTTTCTTTTCACCAGTGAGACGGGCAACAGCTGATTGCCCTTCACCGCCTGGCCCTGAGAGAGTTGCAGCAAGCGGTCCACGCTGGTTTGCCCCAGCAGGCGAAAATCCTGTTTGATGGTGGTTAACGGCGGGATATAACATGAGCTGTCTTCGGTATCGTCGTATCCCACTACCGAGATGTCCGCACCAACGCGCAGCCCGGACTCGGTAATGGCGCGCATTGCGCCCAGCGCCATCTGATCGTTGGCAACCAGCATCGCAGTGGGAACGATGCCCTCATTCAGCATTTGCATGGTTTGTTGAAAACCGGACATGGCACTCCAGTCGCCTTCCCGTTCCGCTATCGGCTGAATTTGATTGCGAGTGAGATATTTATGCCAGCCAGCCAGACGCAGACGCGCCGAGACAGAACTTAATGGGCCCGCTAACAGCGCGATTTGCTGGTGACCCAATGCGACCAGATGCTCCACGCCCAGTCGCGTACCGTCTTCATGGGAGAAAATAATACTGTTGATGGGTGTCTGGTCAGAGACATCAAGAAATAACGCCGGAACATTAGTGCAGGCAGCTTCCACAGCAATGGCATCCTGGTCATCCAGCGGATAGTTAATGATCAGCCCACTGACGCGTTGCGCGAGAAGATTGTGCACCGCCGCTTTACAGGCTTCGACGCCGCTTCGTTCTACCATCGACACCACCACGCTGGCACCCAGTTGATCGGCGCGAGATTTAATCGCCGCGACAATTTGCGACGGCGCGTGCAGGGCCAGACTGGAGGTGGCAACGCCAATCAGCAACGACTGTTTGCCCGCCAGTTGTTGTGCCACGCGGTTGGGAATGTAATTCAGCTCCGCCATCGCCGCTTCCACTTTTTCCCGCGTTTTCGCAGAAACGTGGCTGGCCTGGTTCACCACGCGGGAAACGGTCTGATAAGAGACACCGGCATACTCTGCGACATCGTATAACGTTACTGGTTTCACATTCACCACCCTGAATTGACTCTCTTCCGGGCGCTATCATGCCATACCGCGAAAGGTTTTGCGCCATTCGATGGTGTCCGGGATCTCGACGCTCTCCCTTATGCGACTCCTGCATTAGGAAATTAATACGACTCACTATA | | | | |  |
| Dvl2 DIX WT Dvl2 12-92 |  | | pCDFDuet His6-MBP-TEV | AACAACAACCTCGGGCCGGGTGCGGAAAACCTGTACTTTCAGGGATCCGGCGAGACGAAGGTGATTTACCATCTGGATGAAGAAGAGACTCCTTACCTGGTGAAGATCCCTGTCCCGGCGGAGCGCATCACGCTCGGCGATTTCAAGAGCGTTTTGCAGCGGCCCGCGGGCGCCAAGTACTTTTTCAAGTCCATGGATCAGGATTTTGGGGTGGTGAAGGAAGAGATCTCCGATGACAATGCCCGCCTACCTTGCTTCAATGGAAGGGTTGTCTCCTGGCTTGTGTCGTCATAATAAGTCGAC |  |
| Dvl2 DIX Y27D | Head intra-strand mutant | | pCDFDuet His6-MBP-TEV | AACAACAACCTCGGGCCGGGTGCGGAAAACCTGTACTTTCAGGGATCCGGCGAGACGAAGGTGATTTACCATCTGGATGAAGAAGAGACTCCTGACCTGGTGAAGATCCCTGTCCCGGCGGAGCGCATCACGCTCGGCGATTTCAAGAGCGTTTTGCAGCGGCCCGCGGGCGCCAAGTACTTTTTCAAGTCCATGGATCAGGATTTTGGGGTGGTGAAGGAAGAGATCTCCGATGACAATGCCCGCCTACCTTGCTTCAATGGAAGGGTTGTCTCCTGGCTTGTGTCGTCATAATAAGTCGAC |  |
| Dvl2 DIX K68A | Tail intra-strand mutant | | pCDFDuet His6-MBP-TEV | AACAACAACCTCGGGCCGGGTGCGGAAAACCTGTACTTTCAGGGATCCGGCGAGACGAAGGTGATTTACCATCTGGATGAAGAAGAGACTCCTTACCTGGTGAAGATCCCTGTCCCGGCGGAGCGCATCACGCTCGGCGATTTCAAGAGCGTTTTGCAGCGGCCCGCGGGCGCCAAGTACTTTTTCAAGTCCATGGATCAGGATTTTGGGGTGGTGGCGGAAGAGATCTCCGATGACAATGCCCGCCTACCTTGCTTCAATGGAAGGGTTGTCTCCTGGCTTGTGTCGTCATAATAAGTCGAC |  |
| Dvl2 DIX M60A |  | | pCDFDuet His6-MBP-TEV | AACAACAACCTCGGGCCGGGTGCGGAAAACCTGTACTTTCAGGGATCCGGCGAGACGAAGGTGATTTACCATCTGGATGAAGAAGAGACTCCTTACCTGGTGAAGATCCCTGTCCCGGCGGAGCGCATCACGCTCGGCGATTTCAAGAGCGTTTTGCAGCGGCCCGCGGGCGCCAAGTACTTTTTCAAGTCCGCGGATCAGGATTTTGGGGTGGTGAAGGAAGAGATCTCCGATGACAATGCCCGCCTACCTTGCTTCAATGGAAGGGTTGTCTCCTGGCTTGTGTCGTCATAATAAGTCGAC |  |
| Dvl2 DIX G65D |  | | pCDFDuet His6-MBP-TEV | AACAACAACCTCGGGCCGGGTGCGGAAAACCTGTACTTTCAGGGATCCGGCGAGACGAAGGTGATTTACCATCTGGATGAAGAAGAGACTCCTTACCTGGTGAAGATCCCTGTCCCGGCGGAGCGCATCACGCTCGGCGATTTCAAGAGCGTTTTGCAGCGGCCCGCGGGCGCCAAGTACTTTTTCAAGTCCATGGATCAGGATTTTGATGTGGTGAAGGAAGAGATCTCCGATGACAATGCCCGCCTACCTTGCTTCAATGGAAGGGTTGTCTCCTGGCTTGTGTCGTCATAATAAGTCGAC |  |
| Dvl2 DIX N82D |  | | pCDFDuet His6-MBP-TEV | AACAACAACCTCGGGCCGGGTGCGGAAAACCTGTACTTTCAGGGATCCGGCGAGACGAAGGTGATTTACCATCTGGATGAAGAAGAGACTCCTTACCTGGTGAAGATCCCTGTCCCGGCGGAGCGCATCACGCTCGGCGATTTCAAGAGCGTTTTGCAGCGGCCCGCGGGCGCCAAGTACTTTTTCAAGTCCATGGATCAGGATTTTGGGGTGGTGAAGGAAGAGATCTCCGATGACAATGCCCGCCTACCTTGCTTCGATGGAAGGGTTGTCTCCTGGCTTGTGTCGTCATAATAAGTCGAC |  |
| Dvl2 DIX_DC_ | F64(+DC)G65 | | pCDFDuet His6-MBP-TEV | AACAACAACCTCGGGCCGGGTGCGGAAAACCTGTACTTTCAGGGATCCGGCGAGACGAAGGTGATTTACCATCTGGATGAAGAAGAGACTCCTTACCTGGTGAAGATCCCTGTCCCGGCGGAGCGCATCACGCTCGGCGATTTCAAGAGCGTTTTGCAGCGGCCCGCGGGCGCCAAGTACTTTTTCAAGTCCATGGATCAGGATTTTGACTGTGGGGTGGTGAAGGAAGAGATCTCCGATGACAATGCCCGCCTACCTTGCTTCAATGGAAGGGTTGTCTCCTGGCTTGTGTCGTCATAATAAGTCGAC |  |
| Dvl2 DIX_Y27D/DC_ | Y27D, F64(+DC)G65 | | pCDFDuet His6-MBP-TEV | AACAACAACCTCGGGCCGGGTGCGGAAAACCTGTACTTTCAGGGATCCGGCGAGACGAAGGTGATTTACCATCTGGATGAAGAAGAGACTCCTGACCTGGTGAAGATCCCTGTCCCGGCGGAGCGCATCACGCTCGGCGATTTCAAGAGCGTTTTGCAGCGGCCCGCGGGCGCCAAGTACTTTTTCAAGTCCATGGATCAGGATTTTGACTGTGGGGTGGTGAAGGAAGAGATCTCCGATGACAATGCCCGCCTACCTTGCTTCAATGGAAGGGTTGTCTCCTGGCTTGTGTCGTCATAATAAGTCGAC |  |
| Dvl2 DIX_DE_ | M60(+DE)Q61 | | pCDFDuet His6-MBP-TEV | AACAACAACCTCGGGCCGGGTGCGGAAAACCTGTACTTTCAGGGATCCGGCGAGACGAAGGTGATTTACCATCTGGATGAAGAAGAGACTCCTTACCTGGTGAAGATCCCTGTCCCGGCGGAGCGCATCACGCTCGGCGATTTCAAGAGCGTTTTGCAGCGGCCCGCGGGCGCCAAGTACTTTTTCAAGTCCATGGACGAGGATCAGGATTTTGGGGTGGTGAAGGAAGAGATCTCCGATGACAATGCCCGCCTACCTTGCTTCAATGGAAGGGTTGTCTCCTGGCTTGTGTCGTCATAATAAGTCGAC |  |
| Dvl2 DIX QQ | E22Q, E24Q | | pCDFDuet His6-MBP-TEV | AACAACAACCTCGGGCCGGGTGCGGAAAACCTGTACTTTCAGGGATCCGGCGAGACGAAGGTGATTTACCATCTGGATCAGGAACAGACTCCTTACCTGGTGAAGATCCCTGTCCCGGCGGAGCGCATCACGCTCGGCGATTTCAAGAGCGTTTTGCAGCGGCCCGCGGGCGCCAAGTACTTTTTCAAGTCCATGGATCAGGATTTTGGGGTGGTGAAGGAAGAGATCTCCGATGACAATGCCCGCCTACCTTGCTTCAATGGAAGGGTTGTCTCCTGGCTTGTGTCGTCATAATAAGTCGAC |  |
| Dvl2 DIX KK | E22K, E24K | | pCDFDuet His6-MBP-TEV | AACAACAACCTCGGGCCGGGTGCGGAAAACCTGTACTTTCAGGGATCCGGCGAGACGAAGGTGATTTACCATCTGGATAAAGAAAAGACTCCTTACCTGGTGAAGATCCCTGTCCCGGCGGAGCGCATCACGCTCGGCGATTTCAAGAGCGTTTTGCAGCGGCCCGCGGGCGCCAAGTACTTTTTCAAGTCCATGGATCAGGATTTTGGGGTGGTGAAGGAAGAGATCTCCGATGACAATGCCCGCCTACCTTGCTTCAATGGAAGGGTTGTCTCCTGGCTTGTGTCGTCATAATAAGTCGAC |  |
| Axin1 DAX WT Axin1 743-826 |  | | pCDFDuet His6-MBP-TEV | GAAAACCTGTACTTTCAGGGATCCCCGTGTGACAGCATCGTTGTGGCGTACTACTTCTGCGGGGAACCCATCCCCTACCGCACCCTGGTGAGGGGCCGCGCTGTCACCCTGGGCCAGTTCAAGGAGCTGCTGACCAAAAAGGGCAGCTACAGATACTACTTCAAGAAAGTGAGCGACGAGTTTGACTGTGGGGTGGTGTTTGAGGAGGTTCGAGAGGACGAGGCCGTCCTGCCCGTCTTTGAGGAGAAGATCATCGGCAAAGTGGAGAAGGTGGACTAATAAGTCGAC |  |
| Axin1 DAX Y760D | Head mutant | | pCDFDuet His6-MBP-TEV | GAAAACCTGTACTTTCAGGGATCCCCGTGTGACAGCATCGTTGTGGCGTACTACTTCTGCGGGGAACCCATCCCCGACCGCACCCTGGTGAGGGGCCGCGCTGTCACCCTGGGCCAGTTCAAGGAGCTGCTGACCAAAAAGGGCAGCTACAGATACTACTTCAAGAAAGTGAGCGACGAGTTTGACTGTGGGGTGGTGTTTGAGGAGGTTCGAGAGGACGAGGCCGTCCTGCCCGTCTTTGAGGAGAAGATCATCGGCAAAGTGGAGAAGGTGGACTAATAAGTCGAC |  |
| Axin1 DAX V800A, F801A | Tail mutant | | pCDFDuet His6-MBP-TEV | GGATCCCCGTGTGACAGCATCGTTGTGGCGTACTACTTCTGCGGGGAACCCATCCCCTACCGCACCCTGGTGAGGGGCCGCGCTGTCACCCTGGGCCAGTTCAAGGAGCTGCTGACCAAAAAGGGCAGCTACAGATACTACTTCAAGAAAGTGAGCGACGAGTTTGACTGTGGGGTGGCGGCTGAGGAGGTTCGAGAGGACGAGGCCGTCCTGCC |  |
| Axin1 DAX Y760D, V800A, F801A | Head+Tail mutant | | pCDFDuet His6-MBP-TEV | GGATCCCCGTGTGACAGCATCGTTGTGGCGTACTACTTCTGCGGGGAACCCATCCCCGACCGCACCCTGGTGAGGGGCCGCGCTGTCACCCTGGGCCAGTTCAAGGAGCTGCTGACCAAAAAGGGCAGCTACAGATACTACTTCAAGAAAGTGAGCGACGAGTTTGACTGTGGGGTGGCGGCTGAGGAGGTTCGAGAGGACGAGGCCGTCCTGCCCGTCTTTGAGGAGAAGATCATCGGCAAAGTGGAGAAGGTGGACTAATAAGTCGAC |  |
| Axin1 DAX_NQ/NG_ | D793N, E794Q, E815N, E816G | | pCDFDuet His6-MBP-TEV | GGATCCCCGTGTGACAGCATCGTTGTGGCGTACTACTTCTGCGGGGAACCCATCCCCTACCGCACCCTGGTGAGGGGCCGCGCTGTCACCCTGGGCCAGTTCAAGGAGCTGCTGACCAAAAAGGGCAGCTACAGATACTACTTCAAGAAAGTGAGCAACCAGTTTGACTGTGGGGTGGTGTTTGAGGAGGTTCGAGAGGACGAGGCCGTCCTGCCCGTCTTTAATGGGAAGATCATCGGCAAAGTGGAGAAGGTGGACTAATAAGTCGAC |  |
| Axin1 DAX_NG_ | E815N, E816G | | pCDFDuet His6-MBP-TEV | GGATCCCCGTGTGACAGCATCGTTGTGGCGTACTACTTCTGCGGGGAACCCATCCCCTACCGCACCCTGGTGAGGGGCCGCGCTGTCACCCTGGGCCAGTTCAAGGAGCTGCTGACCAAAAAGGGCAGCTACAGATACTACTTCAAGAAAGTGAGCGACGAGTTTGACTGTGGGGTGGTGTTTGAGGAGGTTCGAGAGGACGAGGCCGTCCTGCCCGTCTTTAATGGGAAGATCATCGGCAAAGTGGAGAAGGTGGACTAATAAGTCGAC |  |
| Axin1 DI-DAX WT 599-826 |  | | pCDFDuet His6-MBP-TEV | GGATCCGGCGTGGCGTGCAAAAGAAATGCCAAGAAGGCTGAGTCGGGGAAGAGCGCCAGCACCGAGGTGCCAGGTGCCTCGGAGGATGCGGAGAAGAACCAGAAAATCATGCAGTGGATCATTGAGGGGGAAAAGGAGATCAGCAGGCACCGCAGGACCGGCCACGGGTCTTCGTGGACGAGGAAGCCACAGCCCCATGAGAACTCCAGACCCTTGTCCCTTGAGCACCCCTGGGCCGGCCCTCAGCTCCGGACCTCCGTGCAGCCCTCCCACCTCTTCATCCAAGACCCCACCATGCCACCCCACCCAGCTCCCAACCCCCTAACCCAGCTGGAGGAGGCGGGCCGACGTCTGGAGGAGGAAGAAAAGAGAGCCAGCCGAGCACCCTCCAAGCAGAGGACAAGATCGCAGAGGAAGGTGGGCGGCGGGAGTGCCCAGCCGTGTGACAGCATCGTTGTGGCGTACTACTTCTGCGGGGAACCCATCCCCTACCGCACCCTGGTGAGGGGCCGCGCTGTCACCCTGGGCCAGTTCAAGGAGCTGCTGACCAAAAAGGGCAGCTACAGATACTACTTCAAGAAAGTGAGCGACGAGTTTGACTGTGGGGTGGTGTTTGAGGAGGTTCGAGAGGACGAGGCCGTCCTGCCCGTCTTTGAGGAGAAGATCATCGGCAAAGTGGAGAAGGTGGACTAATAAGTCGAC |  |
| pGEX-TEV  Sequence with pGEX5 (forward) or pGEX3 (reverse)  ACGTTATCGACTGCACGGTGCACCAATGCTTCTGGCGTCAGGCAGCCATCGGAAGCTGTGGTATGGCTGTGCAGGTCGTAAATCACTGCATAATTCGTGTCGCTCAAGGCGCACTCCCGTTCTGGATAATGTTTTTTGCGCCGACATCATAACGGTTCTGGCAAATATTCTGAAATGAGCTGTTGACAATTAATCATCGGCTCGTATAATGTGTGGAATTGTGAGCGGATAACAATTTCACACAGGAAACAGTATTCATGTCCCCTATACTAGGTTATTGGAAAATTAAGGGCCTTGTGCAACCCACTCGACTTCTTTTGGAATATCTTGAAGAAAAATATGAAGAGCATTTGTATGAGCGCGATGAAGGTGATAAATGGCGAAACAAAAAGTTTGAATTGGGTTTGGAGTTTCCCAATCTTCCTTATTATATTGATGGTGATGTTAAATTAACACAGTCTATGGCCATCATACGTTATATAGCTGACAAGCACAACATGTTGGGTGGTTGTCCAAAAGAGCGTGCAGAGATTTCAATGCTTGAAGGAGCGGTTTTGGATATTAGATACGGTGTTTCGAGAATTGCATATAGTAAAGACTTTGAAACTCTCAAAGTTGATTTTCTTAGCAAGCTACCTGAAATGCTGAAAATGTTCGAAGATCGTTTATGTCATAAAACATATTTAAATGGTGATCATGTAACCCATCCTGACTTCATGTTGTATGACGCTCTTGATGTTGTTTTATACATGGACCCAATGTGCCTGGATGCGTTCCCAAAATTAGTTTGTTTTAAAAAACGTATTGAAGCTATCCCACAAATTGATAAGTACTTGAAATCCAGCAAGTATATAGCATGGCCTTTGCAGGGCTGGCAAGCCACGTTTGGTGGTGGCGACCATCCTCCAAAATCGGATCTGGTTCCGCGTGGATCCATATGTGGTGGCGGAGGGGAAAACCTGTATTTCCAAGGAGGAATTCTAGACTCCATGGGTCGACTCGAGCTCAAGCTTATTCATCGTGACTGACTGACGATCTGCCTCGCGCGTTTCGGTGATGACGGTGAAAACCTCTGACACATGCAGCTCCCGGAGACGGTCACAGCTTGTCTGTAAGCGGATGCCGGGAGCAGACAAGCCCGTCAGGGCGCGTCAGCGGGTGTTGGCGGGTGTCGGGGCGCAGCCATGACCCAGTCACGTAGCGATAGCGGAGTGTATAATTCTTGAAGACGAAAGGGCCTCGTGATACGCCTATTTTTATAGGTTAATGTCATGATAATAATGGTTTCTTAGACGTCAGGTGGCACTTTTCGGGGAAATGTGCGCGGAACCCCTATTTGTTTATTTTTCTAAATACATTCAAATATGTATCCGCTCATGAGACAATAACCCTGATAAATGCTTCAATAATATTGAAAAAGGAAGAGTATGAGTATTCAACATTTCCGTGTCGCCCTTATTCCCTTTTTTGCGGCATTTTGCCTTCCTGTTTTTGCTCACCCAGAAACGCTGGTGAAAGTAAAAGATGCTGAAGATCAGTTGGGTGCACGAGTGGGTTACATCGAACTGGATCTCAACAGCGGTAAGATCCTTGAGAGTTTTCGCCCCGAAGAACGTTTTCCAATGATGAGCACTTTTAAAGTTCTGCTATGTGGCGCGGTATTATCCCGTGTTGACGCCGGGCAAGAGCAACTCGGTCGCCGCATACACTATTCTCAGAATGACTTGGTTGAGTACTCACCAGTCACAGAAAAGCATCTTACGGATGGCATGACAGTAAGAGAATTATGCAGTGCTGCCATAACCATGAGTGATAACACTGCGGCCAACTTACTTCTGACAACGATCGGAGGACCGAAGGAGCTAACCGCTTTTTTGCACAACATGGGGGATCATGTAACTCGCCTTGATCGTTGGGAACCGGAGCTGAATGAAGCCATACCAAACGACGAGCGTGACACCACGATGCCTGCAGCAATGGCAACAACGTTGCGCAAACTATTAACTGGCGAACTACTTACTCTAGCTTCCCGGCAACAATTAATAGACTGGATGGAGGCGGATAAAGTTGCAGGACCACTTCTGCGCTCGGCCCTTCCGGCTGGCTGGTTTATTGCTGATAAATCTGGAGCCGGTGAGCGTGGGTCTCGCGGTATCATTGCAGCACTGGGGCCAGATGGTAAGCCCTCCCGTATCGTAGTTATCTACACGACGGGGAGTCAGGCAACTATGGATGAACGAAATAGACAGATCGCTGAGATAGGTGCCTCACTGATTAAGCATTGGTAACTGTCAGACCAAGTTTACTCATATATACTTTAGATTGATTTAAAACTTCATTTTTAATTTAAAAGGATCTAGGTGAAGATCCTTTTTGATAATCTCATGACCAAAATCCCTTAACGTGAGTTTTCGTTCCACTGAGCGTCAGACCCCGTAGAAAAGATCAAAGGATCTTCTTGAGATCCTTTTTTTCTGCGCGTAATCTGCTGCTTGCAAACAAAAAAACCACCGCTACCAGCGGTGGTTTGTTTGCCGGATCAAGAGCTACCAACTCTTTTTCCGAAGGTAACTGGCTTCAGCAGAGCGCAGATACCAAATACTGTCCTTCTAGTGTAGCCGTAGTTAGGCCACCACTTCAAGAACTCTGTAGCACCGCCTACATACCTCGCTCTGCTAATCCTGTTACCAGTGGCTGCTGCCAGTGGCGATAAGTCGTGTCTTACCGGGTTGGACTCAAGACGATAGTTACCGGATAAGGCGCAGCGGTCGGGCTGAACGGGGGGTTCGTGCACACAGCCCAGCTTGGAGCGAACGACCTACACCGAACTGAGATACCTACAGCGTGAGCTATGAGAAAGCGCCACGCTTCCCGAAGGGAGAAAGGCGGACAGGTATCCGGTAAGCGGCAGGGTCGGAACAGGAGAGCGCACGAGGGAGCTTCCAGGGGGAAACGCCTGGTATCTTTATAGTCCTGTCGGGTTTCGCCACCTCTGACTTGAGCGTCGATTTTTGTGATGCTCGTCAGGGGGGCGGAGCCTATGGAAAAACGCCAGCAACGCGGCCTTTTTACGGTTCCTGGCCTTTTGCTGGCCTTTTGCTCACATGTTCTTTCCTGCGTTATCCCCTGATTCTGTGGATAACCGTATTACCGCCTTTGAGTGAGCTGATACCGCTCGCCGCAGCCGAACGACCGAGCGCAGCGAGTCAGTGAGCGAGGAAGCGGAAGAGCGCCTGATGCGGTATTTTCTCCTTACGCATCTGTGCGGTATTTCACACCGCATAAATTCCGACACCATCGAATGGTGCAAAACCTTTCGCGGTATGGCATGATAGCGCCCGGAAGAGAGTCAATTCAGGGTGGTGAATGTGAAACCAGTAACGTTATACGATGTCGCAGAGTATGCCGGTGTCTCTTATCAGACCGTTTCCCGCGTGGTGAACCAGGCCAGCCACGTTTCTGCGAAAACGCGGGAAAAAGTGGAAGCGGCGATGGCGGAGCTGAATTACATTCCCAACCGCGTGGCACAACAACTGGCGGGCAAACAGTCGTTGCTGATTGGCGTTGCCACCTCCAGTCTGGCCCTGCACGCGCCGTCGCAAATTGTCGCGGCGATTAAATCTCGCGCCGATCAACTGGGTGCCAGCGTGGTGGTGTCGATGGTAGAACGAAGCGGCGTCGAAGCCTGTAAAGCGGCGGTGCACAATCTTCTCGCGCAACGCGTCAGTGGGCTGATCATTAACTATCCGCTGGATGACCAGGATGCCATTGCTGTGGAAGCTGCCTGCACTAATGTTCCGGCGTTATTTCTTGATGTCTCTGACCAGACACCCATCAACAGTATTATTTTCTCCCATGAAGACGGTACGCGACTGGGCGTGGAGCATCTGGTCGCATTGGGTCACCAGCAAATCGCGCTGTTAGCGGGCCCATTAAGTTCTGTCTCGGCGCGTCTGCGTCTGGCTGGCTGGCATAAATATCTCACTCGCAATCAAATTCAGCCGATAGCGGAACGGGAAGGCGACTGGAGTGCCATGTCCGGTTTTCAACAAACCATGCAAATGCTGAATGAGGGCATCGTTCCCACTGCGATGCTGGTTGCCAACGATCAGATGGCGCTGGGCGCAATGCGCGCCATTACCGAGTCCGGGCTGCGCGTTGGTGCGGATATCTCGGTAGTGGGATACGACGATACCGAAGACAGCTCATGTTATATCCCGCCGTTAACCACCATCAAACAGGATTTTCGCCTGCTGGGGCAAACCAGCGTGGACCGCTTGCTGCAACTCTCTCAGGGCCAGGCGGTGAAGGGCAATCAGCTGTTGCCCGTCTCACTGGTGAAAAGAAAAACCACCCTGGCGCCCAATACGCAAACCGCCTCTCCCCGCGCGTTGGCCGATTCATTAATGCAGCTGGCACGACAGGTTTCCCGACTGGAAAGCGGGCAGTGAGCGCAACGCAATTAATGTGAGTTAGCTCACTCATTAGGCACCCCAGGCTTTACACTTTATGCTTCCGGCTCGTATGTTGTGTGGAATTGTGAGCGGATAACAATTTCACACAGGAAACAGCTATGACCATGATTACGGATTCACTGGCCGTCGTTTTACAACGTCGTGACTGGGAAAACCCTGGCGTTACCCAACTTAATCGCCTTGCAGCACATCCCCCTTTCGCCAGCTGGCGTAATAGCGAAGAGGCCCGCACCGATCGCCCTTCCCAACAGTTGCGCAGCCTGAATGGCGAATGGCGCTTTGCCTGGTTTCCGGCACCAGAAGCGGTGCCGGAAAGCTGGCTGGAGTGCGATCTTCCTGAGGCCGATACTGTCGTCGTCCCCTCAAACTGGCAGATGCACGGTTACGATGCGCCCATCTACACCAACGTAACCTATCCCATTACGGTCAATCCGCCGTTTGTTCCCACGGAGAATCCGACGGGTTGTTACTCGCTCACATTTAATGTTGATGAAAGCTGGCTACAGGAAGGCCAGACGCGAATTATTTTTGATGGCGTTGGAATT | | | | |  |
| Axin1 DI-DAX Y760D | Head mutant | | pGEX-TEV | TCGGATCTGGTTCCGCGTGGATCCATATGTGGTGGCGGAGGGGAAAACCTGTATTTCCAAGGAGGAATTCTAGACTCCATGGGTCGACTAGGCGTGGCGTGCAAAAGAAATGCCAAGAAGGCTGAGTCGGGGAAGAGCGCCAGCACCGAGGTGCCAGGTGCCTCGGAGGATGCGGAGAAGAACCAGAAAATCATGCAGTGGATCATTGAGGGGGAAAAGGAGATCAGCAGGCACCGCAGGACCGGCCACGGGTCTTCGGGGACGAGGAAGCCACAGCCCCATGAGAACTCCAGACCCTTGTCCCTTGAGCACCCCTGGGCCGGCCCTCAGCTCCGGACCTCCGTGCAGCCCTCCCACCTCTTCATCCAAGACCCCACCATGCCACCCCACCCAGCTCCCAACCCCCTAACCCAGCTGGAGGAGGCGCGCCGACGTCTGGAGGAGGAAGAAAAGAGAGCCAGCCGAGCACCCTCCAAGCAGAGGACAAGATCGCAGAGGAAGGTGGGCGGCGGGAGTGCCCAGCCGTGTGACAGCATCGTTGTGGCGTACTACTTCTGCGGGGAACCCATCCCCGACCGCACCCTGGTGAGGGGCCGCGCTGTCACCCTGGGCCAGTTCAAGGAGCTGCTGACCAAAAAGGGCAGCTACAGATACTACTTCAAGAAAGTGAGCGACGAGTTTGACTGTGGGGTGGTGTTTGAGGAGGTTCGAGAGGACGAGGCCGTCCTGCCCGTCTTTGAGGAGAAGATCATCGGCAAAGTGGAGAAGGTGGACTGATAGAAGCTTAATTCATCGTGACTGACTGACGATCTGCCTCGCGCGTTTCGGTGATGACG |  |
| pCS2+ M2-Flag  Sequence with Sp6  CGCCATTCTGCCTGGGGACGTCGGAGCAAGCTTGATTTAGGTGACACTATAGAATACAAGCTACTTGTTCTTTTTGCAGGATCCCATCGATTCGAATTCGCCACCATGGACTACAAAGACGATGACGACGACAAGGGACCCATCTTGAGGCCACATTGGGTCTGTGCCCTTGGCCTGGCAAGTTTCAGCCTGGTGCCCTTCATAGTGCCTGGGCTCAGCCTGGCGGCTGGTGCCTACCAAAACGTTTTTGCCACTGTGACTCTCACCAGCAGTGCCTGGTCCTTCCCCCTCTTCCTCCGGGGTAGATGGGGACCTTTGGTTATTTTTAGCTTTATTTTTTATAAGCTTTTTGGGGGGTTAAAATAGACTTTCTTATATTTGGGGGAGTATTTTTTTGTAGATCCAGACATGATAAGATACATTGATGAGTTTGGACAAACCACAACTAGAATGCAGTGAAAAAAATGCTTTATTTGTGAAATTTGTGATGCTATTGCTTTATTTGTAACCATTATAAGCTGCAATAAACAAGTTAACAACAACAATTGCATTCATTTTATGTTTCAGGTTCAGGGGGAGGTGTGGGAGGTTTTTTAATTCGCGGCCGCGGCGCCAATGCATTGGGCCCGGTACCCAGCTTTTGTTCCCTTTAGTGAGGGTTAATTGCGCGCTTGGCGTAATCATGGTCATAGCTGTTTCCTGTGTGAAATTGTTATCCGCTCACAATTCCACACAACATACGAGCCGGAAGCATAAAGTGTAAAGCCTGGGGTGCCTAATGAGTGAGCTAACTCACATTAATTGCGTTGCGCTCACTGCCCGCTTTCCAGTCGGGAAACCTGTCGTGCCAGCTGCATTAATGAATCGGCCAACGCGCGGGGAGAGGCGGTTTGCGTATTGGGCGCTCTTCCGCTTCCTCGCTCACTGACTCGCTGCGCTCGGTCGTTCGGCTGCGGCGAGCGGTATCAGCTCACTCAAAGGCGGTAATACGGTTATCCACAGAATCAGGGGATAACGCAGGAAAGAACATGTGAGCAAAAGGCCAGCAAAAGGCCAGGAACCGTAAAAAGGCCGCGTTGCTGGCGTTTTTCCATAGGCTCCGCCCCCCTGACGAGCATCACAAAAATCGACGCTCAAGTCAGAGGTGGCGAAACCCGACAGGACTATAAAGATACCAGGCGTTTCCCCCTGGAAGCTCCCTCGTGCGCTCTCCTGTTCCGACCCTGCCGCTTACCGGATACCTGTCCGCCTTTCTCCCTTCGGGAAGCGTGGCGCTTTCTCATAGCTCACGCTGTAGGTATCTCAGTTCGGTGTAGGTCGTTCGCTCCAAGCTGGGCTGTGTGCACGAACCCCCCGTTCAGCCCGACCGCTGCGCCTTATCCGGTAACTATCGTCTTGAGTCCAACCCGGTAAGACACGACTTATCGCCACTGGCAGCAGCCACTGGTAACAGGATTAGCAGAGCGAGGTATGTAGGCGGTGCTACAGAGTTCTTGAAGTGGTGGCCTAACTACGGCTACACTAGAAGGACAGTATTTGGTATCTGCGCTCTGCTGAAGCCAGTTACCTTCGGAAAAAGAGTTGGTAGCTCTTGATCCGGCAAACAAACCACCGCTGGTAGCGGTGGTTTTTTTGTTTGCAAGCAGCAGATTACGCGCAGAAAAAAAGGATCTCAAGAAGATCCTTTGATCTTTTCTACGGGGTCTGACGCTCAGTGGAACGAAAACTCACGTTAAGGGATTTTGGTCATGAGATTATCAAAAAGGATCTTCACCTAGATCCTTTTAAATTAAAAATGAAGTTTTAAATCAATCTAAAGTATATATGAGTAAACTTGGTCTGACAGTTACCAATGCTTAATCAGTGAGGCACCTATCTCAGCGATCTGTCTATTTCGTTCATCCATAGTTGCCTGACTCCCCGTCGTGTAGATAACTACGATACGGGAGGGCTTACCATCTGGCCCCAGTGCTGCAATGATACCGCGAGACCCACGCTCACCGGCTCCAGATTTATCAGCAATAAACCAGCCAGCCGGAAGGGCCGAGCGCAGAAGTGGTCCTGCAACTTTATCCGCCTCCATCCAGTCTATTAATTGTTGCCGGGAAGCTAGAGTAAGTAGTTCGCCAGTTAATAGTTTGCGCAACGTTGTTGCCATTGCTACAGGCATCGTGGTGTCACGCTCGTCGTTTGGTATGGCTTCATTCAGCTCCGGTTCCCAACGATCAAGGCGAGTTACATGATCCCCCATGTTGTGCAAAAAAGCGGTTAGCTCCTTCGGTCCTCCGATCGTTGTCAGAAGTAAGTTGGCCGCAGTGTTATCACTCATGGTTATGGCAGCACTGCATAATTCTCTTACTGTCATGCCATCCGTAAGATGCTTTTCTGTGACTGGTGAGTACTCAACCAAGTCATTCTGAGAATAGTGTATGCGGCGACCGAGTTGCTCTTGCCCGGCGTCAATACGGGATAATACCGCGCCACATAGCAGAACTTTAAAAGTGCTCATCATTGGAAAACGTTCTTCGGGGCGAAAACTCTCAAGGATCTTACCGCTGTTGAGATCCAGTTCGATGTAACCCACTCGTGCACCCAACTGATCTTCAGCATCTTTTACTTTCACCAGCGTTTCTGGGTGAGCAAAAACAGGAAGGCAAAATGCCGCAAAAAAGGGAATAAGGGCGACACGGAAATGTTGAATACTCATACTCTTCCTTTTTCAATATTATTGAAGCATTTATCAGGGTTATTGTCTCATGAGCGGATACATATTTGAATGTATTTAGAAAAATAAACAAATAGGGGTTCCGCGCACATTTCCCCGAAAAGTGCCACCTAAATTGTAAGCGTTAATATTTTGTTAAAATTCGCGTTAAATTTTTGTTAAATCAGCTCATTTTTTAACCAATAGGCCGAAATCGGCAAAATCCCTTATAAATCAAAAGAATAGACCGAGATAGGGTTGAGTGTTGTTCCAGTTTGGAACAAGAGTCCACTATTAAAGAACGTGGACTCCAACGTCAAAGGGCGAAAAACCGTCTATCAGGGCGATGGCCCACTACGTGAACCATCACCCTAATCAAGTTTTTTGGGGTCGAGGTGCCGTAAAGCACTAAATCGGAACCCTAAAGGGAGCCCCCGATTTAGAGCTTGACGGGGAAAGCCGGCGAACGTGGCGAGAAAGGAAGGGAAGAAAGCGAAAGGAGCGGGCGCTAGGGCGCTGGCAAGTGTAGCGGTCACGCTGCGCGTAACCACCACACCCGCCGCGCTTAATGCGCCGCTACAGGGCGCGTCCCATTCGCCATTCAGGCTGCGCAACTGTTGGGAAGGGCGATCGGTGCGGGCCTCTTCGCTATTACGCCAGTCGACCATAGCCAATTCAATATGGCGTATATGGACTCATGCCAATTCAATATGGTGGATCTGGACCTGTGCCAATTCAATATGGCGTATATGGACTCGTGCCAATTCAATATGGTGGATCTGGACCCCAGCCAATTCAATATGGCGGACTTGGCACCATGCCAATTCAATATGGCGGACTTGGCACTGTGCCAACTGGGGAGGGGTCTACTTGGCACGGTGCCAAGTTTGAGGAGGGGTCTTGGCCCTGTGCCAAGTCCGCCATATTGAATTGGCATGGTGCCAATAATGGCGGCCATATTGGCTATATGCCAGGATCAATATATAGGCAATATCCAATATGGCCCTATGCCAATATGGCTATTGGCCAGGTTCAATACTATGTATTGGCCCTATGCCATATAGTATTCCATATATGGGTTTTCCTATTGACGTAGATAGCCCCTCCCAATGGGCGGTCCCATATACCATATATGGGGCTTCCTAATACCGCCCATAGCCACTCCCCCATTGACGTCAATGGTCTCTATATATGGTCTTTCCTATTGACGTCATATGGGCGGTCCTATTGACGTATATGGCGCCTCCCCCATTGACGTCAATTACGGTAAATGGCCCGCCTGGCTCAATGCCCATTGACGTCAATAGGACCACCCACCATTGACGTCAATGGGATGGCTCATTGCCCATTCATATCCGTTCTCACGCCCCCTATTGACGTCAATGACGGTAAATGGCCCACTTGGCAGTACATCAATATCTATTAATAGTAACTTGGCAAGTACATTACTATTGGAAGGACGCCAGGGTACATTGGCAGTACTCCCATTGACGTCAATGGCGGTAAATGGCCCGCGATGGCTGCCAAGTACATCCCCATTGACGTCAATGGGGAGGGGCAATGACGCAAATGGGCGTTCCATTGACGTAAATGGGCGGTAGGCGTGCCTAATGGGAGGTCTATATAAGCAATGCTCGTTTAGGGAAC | | | | |  |
| Dvl2 WT | 2-736 | | pCS2+ M2-Flag | GTTACCGCGGGCAGCAGCGCGGGGGGCGGTGGTGTAGGCGAGACGAAGGTGATTTACCATCTGGATGAAGAAGAGACTCCTTACCTGGTGAAGATCCCTGTCCCGGCGGAGCGCATCACGCTCGGCGATTTCAAGAGCGTTTTGCAGCGGCCCGCGGGCGCCAAGTACTTTTTCAAGTCCATGGATCAGGATTTTGGGGTGGTGAAGGAAGAGATCTCCGATGACAATGCCCGCCTACCTTGCTTCAATGGAAGGGTTGTCTCCTGGCTTGTGTCGTCAGATACCCCACAGCCTGAGGTGGCTCCCCCAGCCCATGAGTCTCGGACAGAACTGGTTCCTCCGCCTCCACCCTTACCCCCTTTGCCACCAGAAAGGACCAGTGGAATTGGGGACTCAAGGCCTCCATCCTTCCACCCTAATGTATCCAGCAGCCATGAAAATCTAGAGCCTGAGACAGAGACCGAATCTGTCGTATCACTGAGGCGAGACCGACCTAGGAGGAGAGACAGCAGTGAACATGGCGCTGGTGGCCACAGGCCTGGTGGCCCCTCGAGGCTGGAGCGCCACCTGGCTGGCTACGAGAGCTCTTCCACCCTCATGACCAGTGAGCTGGAGAGTACCAGCCTAGGAGACTCGGATGAGGATGACACCATGAGTAGGTTCAGCAGCTCCACTGAGCAGAGCAGTGCCTCCCGCCTCCTCAAGCGCCACCGAAGGCGAAGGAAGCAGCGGCCGCCACGCATGGAGAGGACCTCATCCTTCAGCAGTGTCACCGATTCCACAATGTCTCTCAACATCATCACGGTCACGCTCAACATGGAGAAGTACAACTTCCTGGGCATCTCCATTGTGGGCCAAAGTAACGAGCGTGGTGATGGGGGCATCTACATCGGCTCCATCATGAAAGGGGGCGCGGTGGCTGCGGACGGGCGGATCGAGCCTGGAGACATGCTTTTGCAGGTAAATGATATGAACTTTGAGAACATGAGCAACGACGATGCTGTACGAGTGCTGAGAGACATCGTGCACAAGCCAGGCCCCATCGTGCTCACCGTGGCCAAGTGTTGGGACCCGTCTCCCCAGGCCTACTTCACCCTCCCTCGAAATGAGCCCATCCAGCCCATCGACCCAGCCGCCTGGGTGTCGCACTCGGCTGCGCTGACTGGAGCCTTCCCTGCTTACCCTGGCTCCTCATCTATGAGCACTATCACATCTGGCTCCTCTCTGCCTGATGGCTGCGAAGGCCGGGGTCTCTCTGTCCACATGGACATGGCCTCTGTCACCAAGGCCATGGCAGCCCCAGAGTCTGGGCTCGAAGTCCGGGACCGCATGTGGCTCAAGATCACCATCCCAAACGCCTTTCTAGGCTCCGATGTGGTGGACTGGCTGTACCATCACGTGGAAGGTTTTCCTGAGCGCCGGGAGGCCCGCAAGTATGCCAGTGGGCTGTTGAAGGCGGGACTCATCCGGCACACCGTCAACAAGATTACTTTCTCTGAGCAGTGCTATTATGTCTTCGGGGACCTCAGTGGTGGCTGTGAGAGTTACCTAGTTAACCTCTCTCTGAATGACAATGACGGTTCCAGTGGGGCTTCAGACCAGGATACCCTGGCACCTCTGCCTGGAGCTACTCCCTGGCCCCTGCTGCCCACCTTCTCCTACCAGTATCCAGCGCCACACCCCTACAGCCCCCAGCCTCCACCCTACCACGAGCTTTCTTCGTACACCTATGGTGGAGGCAGTGCCAGCAGCCAGCACAGTGAGGGGAGCCGGAGCAGCGGGTCGACAAGAAGCGATGGGGGGGCTGGGCGCACAGGCAGGCCTGAGGAACGGGCCCCTGAGTCCAAGTCCGGCAGTGGCAGTGAGTCAGAACTCTCCAGCCGGGGAGGCAGCCTTCGGCGGGGTGGGGAGCCTGGTGGGACTGGTGATGGCGGCCCTCCTCCATCCAGGGGCTCGACAGGCGCTCCTCCTAATCTCCGAGCTCTTCCCGGGCTCCATCCCTATGGAGCCCCATCTGGCATGGCTCTCCCCTATAACCCCATGATGGTAGTTATGATGCCTCCACCCCCACCCCCTGTCTCCACAGCAGTGCAGCCCCCTGGTGCCCCTCCAGTCAGAGACCTGGGCTCCGTGCCCCCAGAACTGACAGCTAGCCGTCAGAGCTTCCACATGGCCATGGGCAACCCCAGTGAGTTTTTTGTGGATGTTATGTAG |  |
| Dvl2 Y27D | Head mutant | | pCS2+ M2-Flag | For more information, see equivalent constructs in MBP-Dvl2 DIX |  |
| Dvl2 K68A | Tail mutant | | pCS2+ M2-Flag | For more information, see equivalent constructs in MBP-Dvl2 DIX |  |
| Dvl2 M60A |  | | pCS2+ M2-Flag | For more information, see equivalent constructs in MBP-Dvl2 DIX |  |
| Dvl2 G65D |  | | pCS2+ M2-Flag | For more information, see equivalent constructs in MBP-Dvl2 DIX |  |
| Dvl2 N82A |  | | pCS2+ M2-Flag | For more information, see equivalent constructs in MBP-Dvl2 DIX |  |
| Dvl2_DC_ | F64(+DC)G65 | | pCS2+ M2-Flag | For more information, see equivalent constructs in MBP-Dvl2 DIX |  |
| Dvl2_DE_ | M60(+DE)Q61 | | pCS2+ M2-Flag | For more information, see equivalent constructs in MBP-Dvl2 DIX |  |
| Dvl2 QQ | E22Q, E24Q | | pCS2+ M2-Flag | For more information, see equivalent constructs in MBP-Dvl2 DIX |  |
| Dvl2 KK | E22K, E24K | | pCS2+ M2-Flag | For more information, see equivalent constructs in MBP-Dvl2 DIX |  |
| Dvl2 ΔDIX | Deletion of Dvl2 residues 2-92 | | pCS2+ M2-Flag | GATACCCCACAGCCTGAGGTGGCTCCCCCAGCCCATGAGTCTCGGACAGAACTGGTTCCTCCGCCTCCACCCTTACCCCCTTTGCCACCAGAAAGGACCAGTGGAATTGGGGACTCAAGGCCTCCATCCTTCCACCCTAATGTATCCAGCAGCCATGAAAATCTAGAGCCTGAGACAGAGACCGAATCTGTCGTATCACTGAGGCGAGACCGACCTAGGAGGAGAGACAGCAGTGAACATGGCGCTGGTGGCCACAGGCCTGGTGGCCCCTCGAGGCTGGAGCGCCACCTGGCTGGCTACGAGAGCTCTTCCACCCTCATGACCAGTGAGCTGGAGAGTACCAGCCTAGGAGACTCGGATGAGGATGACACCATGAGTAGGTTCAGCAGCTCCACTGAGCAGAGCAGTGCCTCCCGCCTCCTCAAGCGCCACCGAAGGCGAAGGAAGCAGCGGCCGCCACGCATGGAGAGGACCTCATCCTTCAGCAGTGTCACCGATTCCACAATGTCTCTCAACATCATCACGGTCACGCTCAACATGGAGAAGTACAACTTCCTGGGCATCTCCATTGTGGGCCAAAGTAACGAGCGTGGTGATGGGGGCATCTACATCGGCTCCATCATGAAAGGGGGCGCGGTGGCTGCGGACGGGCGGATCGAGCCTGGAGACATGCTTTTGCAGGTAAATGATATGAACTTTGAGAACATGAGCAACGACGATGCTGTACGAGTGCTGAGAGACATCGTGCACAAGCCAGGCCCCATCGTGCTCACCGTGGCCAAGTGTTGGGACCCGTCTCCCCAGGCCTACTTCACCCTCCCTCGAAATGAGCCCATCCAGCCCATCGACCCAGCCGCCTGGGTGTCGCACTCGGCTGCGCTGACTGGAGCCTTCCCTGCTTACCCTGGCTCCTCATCTATGAGCACTATCACATCTGGCTCCTCTCTGCCTGATGGCTGCGAAGGCCGGGGTCTCTCTGTCCACATGGACATGGCCTCTGTCACCAAGGCCATGGCAGCCCCAGAGTCTGGGCTCGAAGTCCGGGACCGCATGTGGCTCAAGATCACCATCCCAAACGCCTTTCTAGGCTCCGATGTGGTGGACTGGCTGTACCATCACGTGGAAGGTTTTCCTGAGCGCCGGGAGGCCCGCAAGTATGCCAGTGGGCTGTTGAAGGCGGGACTCATCCGGCACACCGTCAACAAGATTACTTTCTCTGAGCAGTGCTATTATGTCTTCGGGGACCTCAGTGGTGGCTGTGAGAGTTACCTAGTTAACCTCTCTCTGAATGACAATGACGGTTCCAGTGGGGCTTCAGACCAGGATACCCTGGCACCTCTGCCTGGAGCTACTCCCTGGCCCCTGCTGCCCACCTTCTCCTACCAGTATCCAGCGCCACACCCCTACAGCCCCCAGCCTCCACCCTACCACGAGCTTTCTTCGTACACCTATGGTGGAGGCAGTGCCAGCAGCCAGCACAGTGAGGGGAGCCGGAGCAGCGGGTCGACAAGAAGCGATGGGGGGGCTGGGCGCACAGGCAGGCCTGAGGAACGGGCCCCTGAGTCCAAGTCCGGCAGTGGCAGTGAGTCAGAACTCTCCAGCCGGGGAGGCAGCCTTCGGCGGGGTGGGGAGCCTGGTGGGACTGGTGATGGCGGCCCTCCTCCATCCAGGGGCTCGACAGGCGCTCCTCCTAATCTCCGAGCTCTTCCCGGGCTCCATCCCTATGGAGCCCCATCTGGCATGGCTCTCCCCTATAACCCCATGATGGTAGTTATGATGCCTCCACCCCCACCCCCTGTCTCCACAGCAGTGCAGCCCCCTGGTGCCCCTCCAGTCAGAGACCTGGGCTCCGTGCCCCCAGAACTGACAGCTAGCCGTCAGAGCTTCCACATGGCCATGGGCAACCCCAGTGAGTTTTTTGTGGATGTTATGTAG |  |
| DAX-ΔDIX-Dvl2 | Residues 2-92 of Dvl2 substituted wtih Axin1 743-826 | | pCS2+ M2-Flag | CCGTGTGACAGCATCGTTGTGGCGTACTACTTCTGCGGGGAACCCATCCCCTACCGCACCCTGGTGAGGGGCCGCGCTGTCACCCTGGGCCAGTTCAAGGAGCTGCTGACCAAAAAGGGCAGCTACAGATACTACTTCAAGAAAGTGAGCGACGAGTTTGACTGTGGGGTGGTGTTTGAGGAGGTTCGAGAGGACGAGGCCGTCCTGCCCGTCTTTGAGGAGAAGATCATCGGCAAAGTGGAGAAGGTGGACGATACCCCACAGCCTGAGGTGGCTCCCCCAGCCCATGAGTCTCGGACAGAACTGGTTCCTCCGCCTCCACCCTTACCCCCTTTGCCACCAGAAAGGACCAGTGGAATTGGGGACTCAAGGCCTCCATCCTTCCACCCTAATGTATCCAGCAGCCATGAAAATCTAGAGCCTGAGACAGAGACCGAATCTGTCGTATCACTGAGGCGAGACCGACCTAGGAGGAGAGACAGCAGTGAACATGGCGCTGGTGGCCACAGGCCTGGTGGCCCCTCGAGGCTGGAGCGCCACCTGGCTGGCTACGAGAGCTCTTCCACCCTCATGACCAGTGAGCTGGAGAGTACCAGCCTAGGAGACTCGGATGAGGATGACACCATGAGTAGGTTCAGCAGCTCCACTGAGCAGAGCAGTGCCTCCCGCCTCCTCAAGCGCCACCGAAGGCGAAGGAAGCAGCGGCCGCCACGCATGGAGAGGACCTCATCCTTCAGCAGTGTCACCGATTCCACAATGTCTCTCAACATCATCACGGTCACGCTCAACATGGAGAAGTACAACTTCCTGGGCATCTCCATTGTGGGCCAAAGTAACGAGCGTGGTGATGGGGGCATCTACATCGGCTCCATCATGAAAGGGGGCGCGGTGGCTGCGGACGGGCGGATCGAGCCTGGAGACATGCTTTTGCAGGTAAATGATATGAACTTTGAGAACATGAGCAACGACGATGCTGTACGAGTGCTGAGAGACATCGTGCACAAGCCAGGCCCCATCGTGCTCACCGTGGCCAAGTGTTGGGACCCGTCTCCCCAGGCCTACTTCACCCTCCCTCGAAATGAGCCCATCCAGCCCATCGACCCAGCCGCCTGGGTGTCGCACTCGGCTGCGCTGACTGGAGCCTTCCCTGCTTACCCTGGCTCCTCATCTATGAGCACTATCACATCTGGCTCCTCTCTGCCTGATGGCTGCGAAGGCCGGGGTCTCTCTGTCCACATGGACATGGCCTCTGTCACCAAGGCCATGGCAGCCCCAGAGTCTGGGCTCGAAGTCCGGGACCGCATGTGGCTCAAGATCACCATCCCAAACGCCTTTCTAGGCTCCGATGTGGTGGACTGGCTGTACCATCACGTGGAAGGTTTTCCTGAGCGCCGGGAGGCCCGCAAGTATGCCAGTGGGCTGTTGAAGGCGGGACTCATCCGGCACACCGTCAACAAGATTACTTTCTCTGAGCAGTGCTATTATGTCTTCGGGGACCTCAGTGGTGGCTGTGAGAGTTACCTAGTTAACCTCTCTCTGAATGACAATGACGGTTCCAGTGGGGCTTCAGACCAGGATACCCTGGCACCTCTGCCTGGAGCTACTCCCTGGCCCCTGCTGCCCACCTTCTCCTACCAGTATCCAGCGCCACACCCCTACAGCCCCCAGCCTCCACCCTACCACGAGCTTTCTTCGTACACCTATGGTGGTGGAGGCAGTGCCAGCAGCCAGCACAGTGAGGGGAGCCGGAGCAGCGGGTCGACAAGAAGCGATGGGGGGGCTGGGCGCACAGGCAGGCCTGAGGAACGGGCCCCTGAGTCCAAGTCCGGCAGTGGCAGTGAGTCAGAACTCTCCAGCCGGGGAGGCAGCCTTCGGCGGGGTGGGGAGCCTGGTGGGACTGGTGATGGCGGCCCTCCTCCATCCAGGGGCTCGACAGGCGCTCCTCCTAATCTCCGAGCTCTTCCCGGGCTCCATCCCTATGGAGCCCCATCTGGCATGGCTCTCCCCTATAACCCCATGATGGTAGTTATGATGCCTCCACCCCCACCCCCTGTCTCCACAGCAGTGCAGCCCCCTGGTGCCCCTCCAGTCAGAGACCTGGGCTCCGTGCCCCCAGAACTGACAGCTAGCCGTCAGAGCTTCCACATGGCCATGGGCAACCCCAGTGAGTTTTTTGTGGATGTTATGTAG |  |
| DAX Y760D-ΔDIX-Dvl2 | DAX head mutant for DAX-substituted Dvl2 | | pCS2+ M2-Flag | For more information, see equivalent constructs in MBP-Axin1 DAX Y760D |  |
| DAX_NQ/NG_-ΔDIX-Dvl2 | DAX mutations at D793N, E794Q, E815N, E816G for DAX-substituted Dvl2 | | pCS2+ M2-Flag | For more information, see equivalent constructs in MBP-Axin1 DAX_NQ/NG_ |  |
| T2SAM-ΔDIX-Dvl2 | Residues of 2-92 of Dvl2 substituted for human tankyrase2 867-940 | | pCS2+ M2-Flag | GAGAAAAAGGAGGTTCCAGGAGTAGATTTTAGCATAACTCAATTCGTAAGGAATCTTGGACTTGAGCACCTAATGGATATATTTGAGAGAGAACAGATCACTTTGGATGTATTAGTTGAGATGGGGCACAAGGAGCTGAAGGAGATTGGAATCAATGCTTATGGACATAGGCACAAACTAATTAAAGGAGTCGAGAGACTTATCTCCGGACAACAAGGTCTTGATACCCCACAGCCTGAGGTGGCTCCCCCAGCCCATGAGTCTCGGACAGAACTGGTTCCTCCGCCTCCACCCTTACCCCCTTTGCCACCAGAAAGGACCAGTGGAATTGGGGACTCAAGGCCTCCATCCTTCCACCCTAATGTATCCAGCAGCCATGAAAATCTAGAGCCTGAGACAGAGACCGAATCTGTCGTATCACTGAGGCGAGACCGACCTAGGAGGAGAGACAGCAGTGAACATGGCGCTGGTGGCCACAGGCCTGGTGGCCCCTCGAGGCTGGAGCGCCACCTGGCTGGCTACGAGAGCTCTTCCACCCTCATGACCAGTGAGCTGGAGAGTACCAGCCTAGGAGACTCGGATGAGGATGACACCATGAGTAGGTTCAGCAGCTCCACTGAGCAGAGCAGTGCCTCCCGCCTCCTCAAGCGCCACCGAAGGCGAAGGAAGCAGCGGCCGCCACGCATGGAGAGGACCTCATCCTTCAGCAGTGTCACCGATTCCACAATGTCTCTCAACATCATCACGGTCACGCTCAACATGGAGAAGTACAACTTCCTGGGCATCTCCATTGTGGGCCAAAGTAACGAGCGTGGTGATGGGGGCATCTACATCGGCTCCATCATGAAAGGGGGCGCGGTGGCTGCGGACGGGCGGATCGAGCCTGGAGACATGCTTTTGCAGGTAAATGATATGAACTTTGAGAACATGAGCAACGACGATGCTGTACGAGTGCTGAGAGACATCGTGCACAAGCCAGGCCCCATCGTGCTCACCGTGGCCAAGTGTTGGGACCCGTCTCCCCAGGCCTACTTCACCCTCCCTCGAAATGAGCCCATCCAGCCCATCGACCCAGCCGCCTGGGTGTCGCACTCGGCTGCGCTGACTGGAGCCTTCCCTGCTTACCCTGGCTCCTCATCTATGAGCACTATCACATCTGGCTCCTCTCTGCCTGATGGCTGCGAAGGCCGGGGTCTCTCTGTCCACATGGACATGGCCTCTGTCACCAAGGCCATGGCAGCCCCAGAGTCTGGGCTCGAAGTCCGGGACCGCATGTGGCTCAAGATCACCATCCCAAACGCCTTTCTAGGCTCCGATGTGGTGGACTGGCTGTACCATCACGTGGAAGGTTTTCCTGAGCGCCGGGAGGCCCGCAAGTATGCCAGTGGGCTGTTGAAGGCGGGACTCATCCGGCACACCGTCAACAAGATTACTTTCTCTGAGCAGTGCTATTATGTCTTCGGGGACCTCAGTGGTGGCTGTGAGAGTTACCTAGTTAACCTCTCTCTGAATGACAATGACGGTTCCAGTGGGGCTTCAGACCAGGATACCCTGGCACCTCTGCCTGGAGCTACTCCCTGGCCCCTGCTGCCCACCTTCTCCTACCAGTATCCAGCGCCACACCCCTACAGCCCCCAGCCTCCACCCTACCACGAGCTTTCTTCGTACACCTATGGTGGTGGAGGCAGTGCCAGCAGCCAGCACAGTGAGGGGAGCCGGAGCAGCGGGTCGACAAGAAGCGATGGGGGGGCTGGGCGCACAGGCAGGCCTGAGGAACGGGCCCCTGAGTCCAAGTCCGGCAGTGGCAGTGAGTCAGAACTCTCCAGCCGGGGAGGCAGCCTTCGGCGGGGTGGGGAGCCTGGTGGGACTGGTGATGGCGGCCCTCCTCCATCCAGGGGCTCGACAGGCGCTCCTCCTAATCTCCGAGCTCTTCCCGGGCTCCATCCCTATGGAGCCCCATCTGGCATGGCTCTCCCCTATAACCCCATGATGGTAGTTATGATGCCTCCACCCCCACCCCCTGTCTCCACAGCAGTGCAGCCCCCTGGTGCCCCTCCAGTCAGAGACCTGGGCTCCGTGCCCCCAGAACTGACAGCTAGCCGTCAGAGCTTCCACATGGCCATGGGCAACCCCAGTGAGTTTTTTGTGGATGTTATGTAG |  |
| Sm1-ΔDIX-Dvl2 | Residues of 2-92 of Dvl2 substituted for Sm1 1-77 | | pCS2+ M2-Flag | ATGCCGCCTCGGCCACTTGACGTACTTAACAGATCCCTTAAATCTCCCGTGATTGTAAGGCTGAAGGGGGGCCGGGAATTTAGAGGAACGCTGGATGGATATGACATTCACATGAACCTGGTCTTGTTGGACGCCGAGGAGATTCAGAACGGCGAGGTAGTACGAAAGGTCGGCAGCGTAGTAATCAGGGGCGACACTGTAGTGTTTGTAAGTCCTGCCCCAGGTGGAGAAGATACCCCACAGCCTGAGGTGGCTCCCCCAGCCCATGAGTCTCGGACAGAACTGGTTCCTCCGCCTCCACCCTTACCCCCTTTGCCACCAGAAAGGACCAGTGGAATTGGGGACTCAAGGCCTCCATCCTTCCACCCTAATGTATCCAGCAGCCATGAAAATCTAGAGCCTGAGACAGAGACCGAATCTGTCGTATCACTGAGGCGAGACCGACCTAGGAGGAGAGACAGCAGTGAACATGGCGCTGGTGGCCACAGGCCTGGTGGCCCCTCGAGGCTGGAGCGCCACCTGGCTGGCTACGAGAGCTCTTCCACCCTCATGACCAGTGAGCTGGAGAGTACCAGCCTAGGAGACTCGGATGAGGATGACACCATGAGTAGGTTCAGCAGCTCCACTGAGCAGAGCAGTGCCTCCCGCCTCCTCAAGCGCCACCGAAGGCGAAGGAAGCAGCGGCCGCCACGCATGGAGAGGACCTCATCCTTCAGCAGTGTCACCGATTCCACAATGTCTCTCAACATCATCACGGTCACGCTCAACATGGAGAAGTACAACTTCCTGGGCATCTCCATTGTGGGCCAAAGTAACGAGCGTGGTGATGGGGGCATCTACATCGGCTCCATCATGAAAGGGGGCGCGGTGGCTGCGGACGGGCGGATCGAGCCTGGAGACATGCTTTTGCAGGTAAATGATATGAACTTTGAGAACATGAGCAACGACGATGCTGTACGAGTGCTGAGAGACATCGTGCACAAGCCAGGCCCCATCGTGCTCACCGTGGCCAAGTGTTGGGACCCGTCTCCCCAGGCCTACTTCACCCTCCCTCGAAATGAGCCCATCCAGCCCATCGACCCAGCCGCCTGGGTGTCGCACTCGGCTGCGCTGACTGGAGCCTTCCCTGCTTACCCTGGCTCCTCATCTATGAGCACTATCACATCTGGCTCCTCTCTGCCTGATGGCTGCGAAGGCCGGGGTCTCTCTGTCCACATGGACATGGCCTCTGTCACCAAGGCCATGGCAGCCCCAGAGTCTGGGCTCGAAGTCCGGGACCGCATGTGGCTCAAGATCACCATCCCAAACGCCTTTCTAGGCTCCGATGTGGTGGACTGGCTGTACCATCACGTGGAAGGTTTTCCTGAGCGCCGGGAGGCCCGCAAGTATGCCAGTGGGCTGTTGAAGGCGGGACTCATCCGGCACACCGTCAACAAGATTACTTTCTCTGAGCAGTGCTATTATGTCTTCGGGGACCTCAGTGGTGGCTGTGAGAGTTACCTAGTTAACCTCTCTCTGAATGACAATGACGGTTCCAGTGGGGCTTCAGACCAGGATACCCTGGCACCTCTGCCTGGAGCTACTCCCTGGCCCCTGCTGCCCACCTTCTCCTACCAGTATCCAGCGCCACACCCCTACAGCCCCCAGCCTCCACCCTACCACGAGCTTTCTTCGTACACCTATGGTGGTGGAGGCAGTGCCAGCAGCCAGCACAGTGAGGGGAGCCGGAGCAGCGGGTCGACAAGAAGCGATGGGGGGGCTGGGCGCACAGGCAGGCCTGAGGAACGGGCCCCTGAGTCCAAGTCCGGCAGTGGCAGTGAGTCAGAACTCTCCAGCCGGGGAGGCAGCCTTCGGCGGGGTGGGGAGCCTGGTGGGACTGGTGATGGCGGCCCTCCTCCATCCAGGGGCTCGACAGGCGCTCCTCCTAATCTCCGAGCTCTTCCCGGGCTCCATCCCTATGGAGCCCCATCTGGCATGGCTCTCCCCTATAACCCCATGATGGTAGTTATGATGCCTCCACCCCCACCCCCTGTCTCCACAGCAGTGCAGCCCCCTGGTGCCCCTCCAGTCAGAGACCTGGGCTCCGTGCCCCCAGAACTGACAGCTAGCCGTCAGAGCTTCCACATGGCCATGGGCAACCCCAGTGAGTTTTTTGTGGATGTTATGTAG |  |
| Sm1-DIX-Dvl2 | Sm1 residues 1-77 fused to WT Dvl2 | | pCS2+ M2-Flag | ATGCCGCCTCGGCCACTTGACGTACTTAACAGATCCCTTAAATCTCCCGTGATTGTAAGGCTGAAGGGGGGCCGGGAATTTAGAGGAACGCTGGATGGATATGACATTCACATGAACCTGGTCTTGTTGGACGCCGAGGAGATTCAGAACGGCGAGGTAGTACGAAAGGTCGGCAGCGTAGTAATCAGGGGCGACACTGTAGTGTTTGTAAGTCCTGCCCCAGGTGGAGAAGTTACCGCGGGCAGCAGCGCGGGGGGCGGTGGTGTAGGCGAGACGAAGGTGATTTACCATCTGGATGAAGAAGAGACTCCTGACCTGGTGAAGATCCCTGTCCCGGCGGAGCGCATCACGCTCGGCGATTTCAAGAGCGTTTTGCAGCGGCCCGCGGGCGCCAAGTACTTTTTCAAGTCCATGGATCAGGATTTTGGGGTGGTGAAGGAAGAGATCTCCGATGACAATGCCCGCCTACCTTGCTTCAATGGAAGGGTTGTCTCCTGGCTTGTGTCGTCAGATACCCCACAGCCTGAGGTGGCTCCCCCAGCCCATGAGTCTCGGACAGAACTGGTTCCTCCGCCTCCACCCTTACCCCCTTTGCCACCAGAAAGGACCAGTGGAATTGGGGACTCAAGGCCTCCATCCTTCCACCCTAATGTATCCAGCAGCCATGAAAATCTAGAGCCTGAGACAGAGACCGAATCTGTCGTATCACTGAGGCGAGACCGACCTAGGAGGAGAGACAGCAGTGAACATGGCGCTGGTGGCCACAGGCCTGGTGGCCCCTCGAGGCTGGAGCGCCACCTGGCTGGCTACGAGAGCTCTTCCACCCTCATGACCAGTGAGCTGGAGAGTACCAGCCTAGGAGACTCGGATGAGGATGACACCATGAGTAGGTTCAGCAGCTCCACTGAGCAGAGCAGTGCCTCCCGCCTCCTCAAGCGCCACCGAAGGCGAAGGAAGCAGCGGCCGCCACGCATGGAGAGGACCTCATCCTTCAGCAGTGTCACCGATTCCACAATGTCTCTCAACATCATCACGGTCACGCTCAACATGGAGAAGTACAACTTCCTGGGCATCTCCATTGTGGGCCAAAGTAACGAGCGTGGTGATGGGGGCATCTACATCGGCTCCATCATGAAAGGGGGCGCGGTGGCTGCGGACGGGCGGATCGAGCCTGGAGACATGCTTTTGCAGGTAAATGATATGAACTTTGAGAACATGAGCAACGACGATGCTGTACGAGTGCTGAGAGACATCGTGCACAAGCCAGGCCCCATCGTGCTCACCGTGGCCAAGTGTTGGGACCCGTCTCCCCAGGCCTACTTCACCCTCCCTCGAAATGAGCCCATCCAGCCCATCGACCCAGCCGCCTGGGTGTCGCACTCGGCTGCGCTGACTGGAGCCTTCCCTGCTTACCCTGGCTCCTCATCTATGAGCACTATCACATCTGGCTCCTCTCTGCCTGATGGCTGCGAAGGCCGGGGTCTCTCTGTCCACATGGACATGGCCTCTGTCACCAAGGCCATGGCAGCCCCAGAGTCTGGGCTCGAAGTCCGGGACCGCATGTGGCTCAAGATCACCATCCCAAACGCCTTTCTAGGCTCCGATGTGGTGGACTGGCTGTACCATCACGTGGAAGGTTTTCCTGAGCGCCGGGAGGCCCGCAAGTATGCCAGTGGGCTGTTGAAGGCGGGACTCATCCGGCACACCGTCAACAAGATTACTTTCTCTGAGCAGTGCTATTATGTCTTCGGGGACCTCAGTGGTGGCTGTGAGAGTTACCTAGTTAACCTCTCTCTGAATGACAATGACGGTTCCAGTGGGGCTTCAGACCAGGATACCCTGGCACCTCTGCCTGGAGCTACTCCCTGGCCCCTGCTGCCCACCTTCTCCTACCAGTATCCAGCGCCACACCCCTACAGCCCCCAGCCTCCACCCTACCACGAGCTTTCTTCGTACACCTATGGTGGTGGAGGCAGTGCCAGCAGCCAGCACAGTGAGGGGAGCCGGAGCAGCGGGTCGACAAGAAGCGATGGGGGGGCTGGGCGCACAGGCAGGCCTGAGGAACGGGCCCCTGAGTCCAAGTCCGGCAGTGGCAGTGAGTCAGAACTCTCCAGCCGGGGAGGCAGCCTTCGGCGGGGTGGGGAGCCTGGTGGGACTGGTGATGGCGGCCCTCCTCCATCCAGGGGCTCGACAGGCGCTCCTCCTAATCTCCGAGCTCTTCCCGGGCTCCATCCCTATGGAGCCCCATCTGGCATGGCTCTCCCCTATAACCCCATGATGGTAGTTATGATGCCTCCACCCCCACCCCCTGTCTCCACAGCAGTGCAGCCCCCTGGTGCCCCTCCAGTCAGAGACCTGGGCTCCGTGCCCCCAGAACTGACAGCTAGCCGTCAGAGCTTCCACATGGCCATGGGCAACCCCAGTGAGTTTTTTGTGGATGTTATGTAG |  |
| Sm1-DIX_Y27D_-Dvl2 | Sm1 residues 1-77 fused to Dvl2 Y27D | | pCS2+ M2-Flag | For more information, see equivalent constructs in MBP-Dvl2 DIX_Y27D_ |  |
| Sm1-DAX-ΔDIX-Dvl2 | Sm1 residues 1-77 fused to DAX-ΔDIX-Dvl2 | | pCS2+ M2-Flag | ATGCCGCCTCGGCCACTTGACGTACTTAACAGATCCCTTAAATCTCCCGTGATTGTAAGGCTGAAGGGGGGCCGGGAATTTAGAGGAACGCTGGATGGATATGACATTCACATGAACCTGGTCTTGTTGGACGCCGAGGAGATTCAGAACGGCGAGGTAGTACGAAAGGTCGGCAGCGTAGTAATCAGGGGCGACACTGTAGTGTTTGTAAGTCCTGCCCCAGGTGGAGAACCGTGTGACAGCATCGTTGTGGCGTACTACTTCTGCGGGGAACCCATCCCCTACCGCACCCTGGTGAGGGGCCGCGCTGTCACCCTGGGCCAGTTCAAGGAGCTGCTGACCAAAAAGGGCAGCTACAGATACTACTTCAAGAAAGTGAGCGACGAGTTTGACTGTGGGGTGGTGTTTGAGGAGGTTCGAGAGGACGAGGCCGTCCTGCCCGTCTTTGAGGAGAAGATCATCGGCAAAGTGGAGAAGGTGGACGATACCCCACAGCCTGAGGTGGCTCCCCCAGCCCATGAGTCTCGGACAGAACTGGTTCCTCCGCCTCCACCCTTACCCCCTTTGCCACCAGAAAGGACCAGTGGAATTGGGGACTCAAGGCCTCCATCCTTCCACCCTAATGTATCCAGCAGCCATGAAAATCTAGAGCCTGAGACAGAGACCGAATCTGTCGTATCACTGAGGCGAGACCGACCTAGGAGGAGAGACAGCAGTGAACATGGCGCTGGTGGCCACAGGCCTGGTGGCCCCTCGAGGCTGGAGCGCCACCTGGCTGGCTACGAGAGCTCTTCCACCCTCATGACCAGTGAGCTGGAGAGTACCAGCCTAGGAGACTCGGATGAGGATGACACCATGAGTAGGTTCAGCAGCTCCACTGAGCAGAGCAGTGCCTCCCGCCTCCTCAAGCGCCACCGAAGGCGAAGGAAGCAGCGGCCGCCACGCATGGAGAGGACCTCATCCTTCAGCAGTGTCACCGATTCCACAATGTCTCTCAACATCATCACGGTCACGCTCAACATGGAGAAGTACAACTTCCTGGGCATCTCCATTGTGGGCCAAAGTAACGAGCGTGGTGATGGGGGCATCTACATCGGCTCCATCATGAAAGGGGGCGCGGTGGCTGCGGACGGGCGGATCGAGCCTGGAGACATGCTTTTGCAGGTAAATGATATGAACTTTGAGAACATGAGCAACGACGATGCTGTACGAGTGCTGAGAGACATCGTGCACAAGCCAGGCCCCATCGTGCTCACCGTGGCCAAGTGTTGGGACCCGTCTCCCCAGGCCTACTTCACCCTCCCTCGAAATGAGCCCATCCAGCCCATCGACCCAGCCGCCTGGGTGTCGCACTCGGCTGCGCTGACTGGAGCCTTCCCTGCTTACCCTGGCTCCTCATCTATGAGCACTATCACATCTGGCTCCTCTCTGCCTGATGGCTGCGAAGGCCGGGGTCTCTCTGTCCACATGGACATGGCCTCTGTCACCAAGGCCATGGCAGCCCCAGAGTCTGGGCTCGAAGTCCGGGACCGCATGTGGCTCAAGATCACCATCCCAAACGCCTTTCTAGGCTCCGATGTGGTGGACTGGCTGTACCATCACGTGGAAGGTTTTCCTGAGCGCCGGGAGGCCCGCAAGTATGCCAGTGGGCTGTTGAAGGCGGGACTCATCCGGCACACCGTCAACAAGATTACTTTCTCTGAGCAGTGCTATTATGTCTTCGGGGACCTCAGTGGTGGCTGTGAGAGTTACCTAGTTAACCTCTCTCTGAATGACAATGACGGTTCCAGTGGGGCTTCAGACCAGGATACCCTGGCACCTCTGCCTGGAGCTACTCCCTGGCCCCTGCTGCCCACCTTCTCCTACCAGTATCCAGCGCCACACCCCTACAGCCCCCAGCCTCCACCCTACCACGAGCTTTCTTCGTACACCTATGGTGGTGGAGGCAGTGCCAGCAGCCAGCACAGTGAGGGGAGCCGGAGCAGCGGGTCGACAAGAAGCGATGGGGGGGCTGGGCGCACAGGCAGGCCTGAGGAACGGGCCCCTGAGTCCAAGTCCGGCAGTGGCAGTGAGTCAGAACTCTCCAGCCGGGGAGGCAGCCTTCGGCGGGGTGGGGAGCCTGGTGGGACTGGTGATGGCGGCCCTCCTCCATCCAGGGGCTCGACAGGCGCTCCTCCTAATCTCCGAGCTCTTCCCGGGCTCCATCCCTATGGAGCCCCATCTGGCATGGCTCTCCCCTATAACCCCATGATGGTAGTTATGATGCCTCCACCCCCACCCCCTGTCTCCACAGCAGTGCAGCCCCCTGGTGCCCCTCCAGTCAGAGACCTGGGCTCCGTGCCCCCAGAACTGACAGCTAGCCGTCAGAGCTTCCACATGGCCATGGGCAACCCCAGTGAGTTTTTTGTGGATGTTATGTAG |  |
| Sm1-DAX Y760D-ΔDIX-Dvl2 | Sm1 residues 1-77 fused to DAX Y760D-ΔDIX-Dvl2 | | pCS2+ M2-Flag | For more information, see equivalent constructs in MBP-Axin1 DAX_Y760D_ |  |
| Sm1 |  | |  | Sm1 amino acid sequence: MPPRPLDVLNRSLKSPVIVRLKGGREFRGTLDGYDIHMNLVLLDAEEIQNGEVVRKVGSVVIRGDTVVFVSPAPGGE  Minigene from IDT (codon optimized for human cells):  ATGCCGCCTCGGCCACTTGACGTACTTAACAGATCCCTTAAATCTCCCGTGATTGTAAGGCTGAAGGGGGGCCGGGAATTTAGAGGAACGCTGGATGGATATGACATTCACATGAACCTGGTCTTGTTGGACGCCGAGGAGATTCAGAACGGCGAGGTAGTACGAAAGGTCGGCAGCGTAGTAATCAGGGGCGACACTGTAGTGTTTGTAAGTCCTGCCCCAGGTGGAGAA |  |
